# Meat-cooking mutagens and risk of renal cell carcinoma

**DOI:** 10.1038/bjc.2011.343

**Published:** 2011-09-06

**Authors:** C R Daniel, K L Schwartz, J S Colt, L M Dong, J J Ruterbusch, M P Purdue, A J Cross, N Rothman, F G Davis, S Wacholder, B I Graubard, W H Chow, R Sinha

**Affiliations:** 1 Division of Cancer Epidemiology and Genetics, National Cancer Institute, National Institutes of Health, 6120 Executive Boulevard, Rockville, MD 20852, USA; 2 nos Cancer Institute, Wayne State University, 110 East Warren, Detroit, MI 48201, USA; 3 Division of Epidemiology and Biostatistics, School of Public Health, University of Illinois, 877 SOHOIMIC 923, 1603 West Taylor Street, Chicago, IL 60612, USA; 4 Department of Family Medicine and Public Health Sciences, Wayne State University, 110 East Warren, Detroit, MI 48201, USA

**Keywords:** benzo(a)pyrene, heterocyclic amines, meat intake, African Americans, smoking, kidney cancer

## Abstract

**Background:**

High-temperature cooked meat contains two families of carcinogens, heterocyclic amines (HCAs) and polycyclic aromatic hydrocarbons (PAHs). Given the kidneys’ role in metabolism and urinary excretion of these compounds, we investigated meat-derived mutagens, as well as meat intake and cooking methods, in a population-based case–control study conducted in metropolitan Detroit and Chicago.

**Methods:**

Newly diagnosed, histologically confirmed adenocarcinoma of the renal parenchyma (renal cell carcinoma (RCC)) cases (*n*=1192) were frequency matched on age, sex, and race to controls (*n*=1175). The interviewer-administered Diet History Questionnaire (DHQ) included queries for meat-cooking methods and doneness with photographic aids. Levels of meat mutagens were estimated using the DHQ in conjunction with the CHARRED database.

**Results:**

The risk of RCC increased with intake of barbecued meat (*P*_trend_=0.04) and the PAH, benzo(*a*)pyrene (BaP) (multivariable-adjusted odds ratio and 95% confidence interval, highest *vs* lowest quartile: 1.50 (1.14, 1.95), *P*_trend_=0.001). With increasing BaP intake, the risk of RCC was more than twofold in African Americans and current smokers (*P*_interaction_<0.05). We found no association for HCAs or overall meat intake.

**Conclusion:**

BaP intake, a PAH in barbecued meat, was positively associated with RCC. These biologically plausible findings advocate further epidemiological investigation into dietary intake of BaP and risk of RCC.

Renal cell carcinoma (RCC), the most common kidney neoplasm in adults, originates in the lining of the proximal convoluted tubule, where glucose, amino acids, uric acid, and inorganic salts are reabsorbed into the filtrate (Barjorin, 2007). Renal cell carcinoma is more common in men than women, and the rates remain higher in African Americans (Chow *et al*, 2010; Jemal *et al*, 2010). Despite associations with diet-related chronic conditions, such as obesity, hypertension, and diabetes, dietary risk factors for RCC are not well established (World Cancer Research Fund/American Institute for Cancer Research, 2007; Chow *et al*, 2010). Intake of fruits and vegetables may lower RCC risk (Rashidkhani *et al*, 2005; Lee *et al*, 2006), whereas high meat intake may increase risk (Faramawi *et al*, 2007; Aune *et al*, 2009; Dolwick Grieb *et al*, 2009), but the epidemiological evidence for various aspects of diet is largely inconsistent.

Cooking of meat at high temperatures may result in the formation and ingestion of carcinogenic compounds, including heterocyclic amines (HCAs) and polycyclic aromatic hydrocarbons (PAHs) (Rothman *et al*, 1993; Phillips, 1999; Sinha and Norat, 2002). Heterocyclic amines, the pyrolysis products of amino acids and creatine/creatinine, are formed in the meat's fatty juices upon direct contact with a high-heat cooking surface, such as during pan-frying (Sinha *et al*, 1998). Meat cooked above an open flame or embers, as with barbecuing or grilling, contains the highest levels of PAHs in the smoke formed as the meat's fatty juices drip onto the heat source (Kazerouni *et al*, 2001).

Dietary intake of HCAs and benzo(*a*)pyrene (BaP), a PAH found in the diet (Rothman *et al*, 1993; Kazerouni *et al*, 2001), have been linked to tumour formation at multiple organ sites in animal feeding studies (Nagao and Sugimura, 1993; Sinha and Norat, 2002). These compounds have also been associated with cancers of the colorectum (Sinha *et al*, 2005b; Cross *et al*, 2010), lung (Lam *et al*, 2009; Tasevska *et al*, 2009), pancreas (Anderson *et al*, 2005; Stolzenberg-Solomon *et al*, 2007), prostate (Cross *et al*, 2005), and breast (Sinha *et al*, 2000) in several large epidemiological investigations; however, results across studies and cancer sites are not consistent (Alaejos *et al*, 2008; Mignone *et al*, 2009; Tasevska *et al*, 2010; Wu *et al*, 2010). Despite the kidneys’ role in metabolism and urinary excretion of these compounds (Turteltaub *et al*, 1997; Peters *et al*, 2004), human investigations of meat mutagens and kidney cancer are sparse and limited by small numbers of cases (Lindblad *et al*, 1997; De Stefani *et al*, 1998; Augustsson *et al*, 1999), narrow range of intake (Augustsson *et al*, 1999), and/or lack of comprehensive assessments of cooking methods and doneness to calculate the levels of potential meat-cooking carcinogens (Wolk *et al*, 1996; Lindblad *et al*, 1997). We investigated intake of meat and meat-related mutagens in relation to RCC in a large, population-based case–control study of US men and women who completed a Diet History Questionnaire (DHQ) with a meat-cooking module. We also examined whether these associations varied by race, gender, or smoking status.

## Materials and methods

The Kidney Cancer Study (United States), a population-based case–control study conducted in metropolitan Detroit and Chicago, is described elsewhere (Karami *et al*, 2010). Briefly, Caucasian (CA) and African American (AA) men and women between 20 and 79 years of age were eligible for the study. Cases were comprised of newly diagnosed, histologically confirmed adenocarcinoma of the renal parenchyma (RCC (ICD-O2-C64.9)). Cases were ascertained through the Metropolitan Detroit Cancer Surveillance System (Surveillance Epidemiology and End Results (SEER) program member) between February 2002 and July 2006 (CAs) or January 2007 (AAs), and through review of pathology reports for cases diagnosed in 2003 at Cook County, Chicago Hospitals. Community controls were selected from the Department of Motor Vehicle (DMV) identification records (aged 20–64 years) and Medicare beneficiary files (aged 65 years and older), and frequency-matched to cases on age (at 5-year intervals), sex, race, and centre. The sampling strategy employed to increase the number of AA participants, as well as recruitment and participation rates, is described in detail elsewhere (DiGaetano *et al*, 2003; Karami *et al*, 2010; Li *et al*, 2011). Briefly, of the 1918 cases identified and 2718 presumed eligible controls, 341 cases and 449 controls could not be located. Of the 1571 cases we sought to enrol, 1217 cases (77.5%) entered the study (221 declined participation and 133 were not interviewed owing to serious illness, impairment, or failure to respond to multiple contact attempts). Of the 2269 controls we attempted to recruit, 677 declined participation and 357 were unable to be interviewed; thus, 54.4% (1235 controls) participated.

Trained staff administered an in-home, computer-assisted interview to elicit information from participants on demographics, use of tobacco and alcohol, diet (described in detail below), height and weight history, family history of cancer, and medical history. All participants provided written informed consent and study centres were approved by their respective human subjects review boards.

Participants were excluded from the present diet analysis if they did not complete the DHQ (8 cases, 11 controls); reported extreme or implausible values for total non-alcohol energy intake (apparent outliers in the top and bottom 1% of the control distribution: <600 kcal or >6000 kcal; 19 cases, 18 controls); or if the interviewer noted issues with the participant's cognitive recall and/or conflicting responses to repetitive sequences (15 cases, 14 controls). This resulted in a final sample of 1175 cases and 1192 controls.

### Dietary assessment

The modified DHQ collected information on usual adult diet 2 years before the reference date (date of diagnosis for cases or date of sample selection for controls) (Mares-Perlman *et al*, 1993; Willett, 1998; Subar *et al*, 2001). The DHQ was interviewer-administered and queried the frequency of intake of a wide range of foods (cereals and grains, fruits, vegetables, legumes, meats, dairy, desserts, fast foods), beverages (water, non-alcoholic, and alcoholic), and vitamin supplements. Each section included additional questions to collect details on the way the food was usually consumed (e.g., raw, cooked, canned, or pickled vegetables; chicken with or without skin; fried, baked, or mashed potatoes) and items typically added to food (e.g., milk or sugar to coffee or cereal, fat or meat used to cook or flavour greens, salad dressing, gravy, butter, syrup). For beverages, the number of glasses or cans was recorded. However, to decrease respondent burden and the potential for error, standard portion sizes were used to derive gram values for food items (Subar *et al*, 2000).

The following meat, poultry, and fish items were queried and mutually exclusive variables created: processed meat (red processed: bacon, sausage, ham hocks/smoked pork added to greens; and mixed processed: deli meat/cold cuts, hot dogs); red meat, not processed (beef: hamburger, ground beef/beef stew, roast beef/pot roast, beef steak; and pork: ham/ham-steak, pork chops, pork roast, pork/beef ribs); and white meat (poultry: chicken, turkey, fried chicken, ground chicken/turkey; and fish: canned/smoked/salted fish, fresh fish/seafood, fried fish). Standard fractions of meat components of mixed dishes such as pizza, pasta, and chilli/stew (USDA, 2008) were also included as a ‘red meat mix’ variable.

The meat portion of the DHQ included a meat-cooking module (Cantwell *et al*, 2004; Sinha *et al*, 2005a) with corresponding queries for cooking method (grilled or barbecued, baked or roasted, oven-broiled, breaded and fried, pan-fried without breading, boiled or stewed, microwaved, brown n’ serve, other, don’t know). Photographic models were used to help participants select a level of doneness (inside and outside) on an incremental scale (rare, medium, medium-well, well, very-well done) (Sinha and Rothman, 1997; Anderson *et al*, 2005). Frequency of meat intake, usual cooking method, and level of doneness were linked to the ‘computerized HCA resource for research in epidemiology of disease’ (CHARRED; http://charred.cancer.gov/) database of measured mutagenicity in cooked meats to estimate values (ng per day) of three HCAs (2-amino-1-methyl-6-phenyl-imidazo(4,5-*b*)pyridine (PhIP), 2-amino-3,8-dimethylimidazo(4,5-*f*)quinoxaline (MeIQx), 2-amino-3,4,8-trimethylimidazo(4,5-*f*)quinoxaline (DiMeIQx)), and one PAH (BaP) using an exposure index described in detail elsewhere (Kazerouni *et al*, 2001; Sinha *et al*, 2005a). Correlation coefficients between the DHQ with meat-cooking module and multiple food diaries ranged from 0.31 to 0.46 for chicken, red, and grilled meat intake; and ranged from 0.36 to 0.60 for meat-mutagen intake (Cantwell *et al*, 2004).

### Statistical analysis

A set of sample weights were developed to reduce the potential for bias arising from differential sampling rates for controls and cases, from survey non-response, and from deficiencies in coverage of the population at-risk by the DMV and Medicare files used to select controls (Li *et al*, 2011). Sample weights for controls also included a post-stratification adjustment, so that the weighted distribution of controls across the matching variables exactly matched the weighted distribution of cases. In addition to being consistent with the objectives of the frequency matching, the post-stratification adjustment reduces the variability of the weights compared to not using this adjustment (Li *et al*, 2011).

Foods and nutrients were standardised (g per 1000 kcal) for total energy intake, excluding energy from alcohol (non-alcohol energy intake). All continuous exposure variables were categorised into quartiles based on the distributions among controls, with the referent group comprised of individuals in the lowest category of intake. If a variable had a large proportion (e.g., two-thirds to three-quarters) of zero values, then categories were created by setting all zero values to the referent group and categorising the non-zero values into tertiles (as shown in Table 3 for barbecued and broiled meat). To evaluate a wider range of intake and potentially skewed distributions for the meat mutagens (Augustsson *et al*, 1999; Sinha, 2002), we additionally investigated associations across deciles for BaP, DiMeIQx, MeIQx, and PhIP.

We estimated the risk of developing RCC by deriving adjusted odds ratios (ORs) and 95% confidence intervals (CIs) from unconditional logistic regression models using post-stratified weights. Jackknife replicate weights were created to estimate standard errors (Rust and Rao, 1996). Model covariates (categories defined in Table 1) included study site, age group, self-reported race, sex, education, smoking history as of 2 years before interview, body mass index (BMI, based on height at interview and weight 5 years before interview), history of cancer among first-degree relatives, diagnosis of hypertension 2 or more years before the reference date (self-reported), fruit and vegetable intake (MyPyramid equivalents (Friday and Bowman, 2006); modelled in quartiles), and alcohol intake (quartiles). All models included a continuous covariate for non-alcohol energy intake and were additionally adjusted for other meat variables (e.g., red meat intake adjusted for white meat intake, barbecued meat intake adjusted for intake of meat cooked by other methods, and so on). We also considered potential confounding by other dietary factors, including whole grains and fats, mutual adjustment for meat mutagens (e.g., BaP adjusted for HCAs), as well as energy adjustment, including alcohol intake. We also conducted an unweighted analysis (i.e., not weighting by the post-stratified sample weights) using standard logistic regression procedures, but none of these modifications produced results dissimilar to those presented (data not shown). Tests for trend were performed by creating a continuous variable from the median value for intake in controls within each quartile.

We evaluated effect modification by smoking status, gender, race, BMI, and history of hypertension, by including product terms of the levels of dietary intake (quartiles) and the levels of the factor (i.e., 2 for race and 3 for smoking) in the multiple logistic regression model and testing for the joint significance of the additional terms using the Wald *χ*^2^ test that is appropriate for weighted data (Korn and Graubard, 1999). Statistical tests were determined to be significant at a two-sided *P*<0.05. All analyses were conducted with the SAS software version 9.2 (SAS Institute Inc., Cary, NC, USA) using procedures appropriate for sample-weighted data (SAS Institute, 2008).

## Results

Cases tended to be less educated than controls, and they were more likely to be obese, to have a history of hypertension, and to be a current smoker (Table 1). Distributions of demographic and lifestyle characteristics were similar by race (shown in Karami *et al*, 2010). Intake of red meat, barbecued meat, BaP, and alcohol were significantly higher in cases compared to controls, whereas poultry intake was significantly lower (all *P*_diff_ <0.01; *P*-values not shown). By race, MeIQx was significantly higher in CA cases compared to CA controls, whereas alcohol, red, and processed meat intake was significantly higher in AA cases compared to AA controls.

Correlations between the mutually exclusive meat-related exposures investigated in the analysis are presented in Table 2. BaP was positively correlated with non-processed red meat (*r*=0.47) and barbecued meat (*r*=0.73) intake, whereas HCAs (DiMeIQx, MeIQx, and PhIP) were more strongly correlated with pan-fried meat intake (*r*=0.55–0.77). In this study population, majority of the BaP and HCA intake (70–90%) came from red meat sources, as compared to white meat sources (data not shown).

In multivariable-adjusted models (Table 3), we found a statistically significant excess risk of RCC for those in the highest, compared to the lowest, intake quartile of barbecued meat and BaP. When mutually adjusted for intake of the HCAs (data not shown), the OR and 95% CI for highest to lowest quartile of BaP was 1.84 (1.37, 2.49). Conversely, high intake of broiled meat, when adjusted for confounders and intake of meat cooked by other methods, was associated with lower risk of RCC. We found no association for pan-fried meat, or for baked, microwaved, and stewed meat (data not shown). Nor did we find associations for any of the HCAs. In addition to the null findings for total, red, and white meat, we found no association for intake of fish (or subgroups of fresh, fried, or canned/salted/smoked), poultry, processed, or fresh red meat (data not shown). Although much of the white meat intake in this population was cooked by methods that do not generate BaP or HCAs (e.g., baking or roasting; see Supplementary Table S1), we observed similar associations for meat mutagens from red *vs* white meat sources when comparing equivalent levels of intake (OR and 95% CI, per 5 ng per day of BaP intake: 1.01 (1.00–1.02) and 1.02 (0.99–1.05) from red and white meat, respectively). We found a suggestive positive association for ‘smoke-cured red meat’ (sum of bacon, sausage, and pork added to greens), a proxy for processed meats with higher BaP content (Kazerouni *et al*, 2001; Lee and Shim, 2007; Reinik *et al*, 2007): covariate-adjusted OR and 95% CI for highest *vs* lowest quartile (1.24 (0.95, 1.62), data not shown).

We found a statistically significant interaction by race (Table 4) for BaP (*P*_BaP × race_=0.05), as well as for broiled meat intake (*P*_broil × race_=0.03). For broiled meat, we observed an inverse association for the highest *vs* lowest quartile of intake among AAs (OR and 95% CI: 0.55 (0.35, 0.85)). Among CA participants, the association with broiled meat was attenuated (OR=0.84, 95% CI=0.62–1.15). In AAs, we observed a significant increase in RCC risk for both the third (1.77 (1.10, 2.83)) and fourth (2.00 (1.15, 3.47)) quartile of BaP intake, respectively. In CAs, an increase in risk (1.42 (1.04, 1.93)) was only detectable in the fourth quartile (median intake, 61 ng per day).

The association between BaP and RCC also differed by smoking status (Table 5; *P*_BaP × smoke_=0.02). In the third and fourth quartile of intake (median, 12 and 53 ng per day, respectively), the risk of RCC associated with BaP more than doubled in current-regular smokers. The corresponding risk among never smokers was 1.47 (0.93, 2.31) in the highest, compared to lowest, quartile. We found no evidence of effect modification by smoking status for any of the HCAs (all *P*_interaction_>0.8) and no other evidence of interaction between meat mutagens, or for any of the other meat exposures with race, gender, BMI, history of hypertension, or smoking status. Results did not materially differ in analyses restricted to the primary histomorphological subtype, clear cell RCC (71% of total cases).

## Discussion

In this large, population-based case–control study with a detailed diet history and meat-cooking module with photographic aids, we found that estimated intake of BaP from meat, a carcinogen found in the smoke formed during high-temperature cooking such as barbecuing, was associated with a 50% increased risk of RCC in cases compared with controls. In AA participants, BaP was associated with a more than twofold increase in risk, whereas broiled meat intake was inversely associated with RCC. The positive association between BaP and RCC was also more pronounced in current-regular smokers. We did not find any significant associations with RCC across a wide range of HCA intakes.

Experimental evidence suggests that exposure to HCA and PAH metabolites, ingested in the diet and processed by the kidney through the course of metabolism, may be a biologically plausible risk factor for kidney cancer (Adamson *et al*, 1990; Thorgeirsson *et al*, 1995; Turteltaub *et al*, 1997; Shimada *et al*, 2003; Peters *et al*, 2004; Marie *et al*, 2010). The proximal convoluted tubule, where RCC forms in the epithelial lining, may be particularly susceptible to disease owing to its early exposure to toxins in the glomerular filtrate before urinary excretion (Barjorin, 2007). Nevertheless, few epidemiological studies of kidney cancer have evaluated dietary intake of these carcinogenic compounds (De Stefani *et al*, 1998; Augustsson *et al*, 1999) and we are the first to report an association for dietary intake of BaP and risk of RCC, as well as differential associations by race and smoking status. In previous epidemiological studies, BaP intake from high-temperature cooked meat has been associated with cancers of the colorectum (Gunter *et al*, 2005; Sinha *et al*, 2005b), pancreas (Anderson *et al*, 2005; Li *et al*, 2007), and lung (Lam *et al*, 2009). A small case–control study in Uruguay reported that barbecued meat and HCA intakes were associated with a significant increased risk of RCC (De Stefani *et al*, 1998). A larger, multicentre, international case–control study of RCC (Wolk *et al*, 1996) reported similar findings for ‘well done or charred’ usual degree of meat doneness (proxy for HCAs and/or PAHs). Two case–control studies (Wolk *et al*, 1996; Lindblad *et al*, 1997) reported a positive association for meat that was fried or sautéed, a general proxy for HCAs. Similar to our null results for pan-fried meat and HCAs, a Swedish population-based case–control study also found no association between HCAs and kidney cancer, but postulated that this may be due to low exposure or narrow range of intake (Augustsson *et al*, 1999). We examined more extreme levels (10th and 90th percentiles) of HCA intake and still did not find an association.

Racial differences in cooking practices, such as a well-done meat preference and thus greater exposure to HCAs among AA, have been documented (Bogen and Keating, 2001; Keating and Bogen, 2004). Although in our population AAs consumed greater amounts of pan-fried meat and HCAs than CA participants, we found no association with risk of RCC in either race. Differential findings for BaP by race do not appear to be explained by differences in overall intake, but we cannot rule out the possibility of residual confounding by other divergent risk factors in AA and CA participants. There is some evidence that genetic mutations in the *CYP1A1* gene are more common in AAs (Crofts *et al*, 1993; Nock *et al*, 2007), whose product, aromatic hydrocarbon hydroxylase, catalyses the first step in the conversion of BaP to a DNA-binding carcinogen. Additional research in diverse populations is needed to clarify these findings.

In addition to dietary sources (Rothman *et al*, 1993; Kazerouni *et al*, 2001), inhalation of cigarette smoke, air pollution, and occupational exposures to combustion by-products may also contribute to BaP exposure (Butler *et al*, 1993; Alexandrov *et al*, 2010; Golka *et al*, 2004). As cigarette smoke contains BaP (Ding *et al*, 2005) and smoking is a known risk factor for kidney cancer (Chow *et al*, 2010), we also investigated whether smoking status modified the association between RCC and dietary intake of BaP from cooked meat. We found more pronounced risk at lower levels of intake among current-regular smokers compared to never smokers. The effect of smoking may be additive, that is, BaP in cigarette smoke plus dietary intake from meat results in higher overall BaP exposure and thus higher risk, and/or smoking may increase the formation of DNA adducts through upregulated conversion of BaP to benzo(*a*)pyrene-7,8-diol-9,10-epoxide via cytochrome *P*450 enzymes (Rubin, 2001; Alexandrov *et al*, 2010). In the general non-smoking population without substantial exposure to occupational hazards and severe pollution, the diet is likely to be the largest source of BaP exposure (i.e., approximately 70–96% of total exposure in non-smokers) (Lioy and Greenberg, 1990; Menzie *et al*, 1992; Butler *et al*, 1993).

Consistent with the current epidemiological literature for meat and RCC (Lee *et al*, 2008; Alexander and Cushing, 2009), we found no association for total meat or any individual meat types across our study population; thus, it appears that the cooking method, rather than overall meat intake, may be important. Oven-broiling generally results in the formation of little or no meat-cooking compounds (Kazerouni *et al*, 2001; Sinha, 2002), and in our population was associated with lower risk of RCC in AAs, as well as inversely correlated with barbecued meat intake. In a similar investigation of meat-cooking exposures and colorectal neoplasia, broiled meat intake appeared protective, whereas intake of barbecued meat and BaP was associated with higher risk (Gunter *et al*, 2005). In our study, intake of ‘smoke-cured’ meat, which is expected to contribute to BaP intake (Garcia Falcon *et al*, 1999; Kazerouni *et al*, 2001; Lee and Shim, 2007; Reinik *et al*, 2007), also showed a suggestive positive association with RCC. Red and processed meat may also be a source of *N*-nitroso compounds (Joosen *et al*, 2009), which have been shown to induce renal tumours in rodent models (Hard, 1998). Detailed analyses of these compounds, which are also found in plant foods and drinking water, are ongoing.

The interviewer-administered DHQ with meat-cooking module (Cantwell *et al*, 2004; Sinha *et al*, 2005a) was a strength of our study. Although we cannot rule out the possibility of measurement error, photographic aids were used in this study to help the participant select a level of doneness on an incremental scale. Other studies have asked participants to recall the usual appearance of cooked meat without a visual or objective reference and/or did not measure BaP. Additional strengths of our study include a wide range of intake (possibly due to the diversity of our sample), histologically confirmed RCC cases, a relatively large sample size, and adjustment for a multitude of potential confounders, including self-reported history of hypertension, BMI, and smoking status. On the other hand, data collection in this study was based entirely on self-report, and thus subject to recall bias, interviewer bias, differential reporting among cases and controls, and/or non-differential misclassification. However, given the lack of well-established dietary risk factors for RCC and the use of computer-based, structured questionnaires by trained interviewers, it is unlikely that differential recall or interviewer bias had a strong impact on our findings. We did not have the means to calculate relevant BaP intake from other foods for this population; thus, we might have underestimated and/or misclassified categories of BaP from diet in both cases and controls. Other limitations of population-based studies also apply, including selection and non-response bias, which may affect the internal and external validity of our findings. The creation of sample weights using demographic data from both respondents and non-respondents and use of weighted analytic procedures aims to reduce biases arising from low response rates among controls and differential non-response among subgroups (age, sex, race, residence) (Li *et al*, 2011). We cannot rule out the possibility of chance findings due to multiple comparisons. Statistically significant findings close to the nominal level (*α*=0.05) should be viewed with caution.

BaP, a PAH found in barbecued meat, was associated with higher risk of RCC. Beyond *in vivo* and animal experimental evidence, findings from occupational and environmental health studies for PAHs and cancer risk (Butler *et al*, 1993; Mandel *et al*, 1995; Pesch *et al*, 2000; Spinelli *et al*, 2006) combined with epidemiological findings for dietary BaP from meat and other cancers (Anderson *et al*, 2005; Sinha *et al*, 2005b) lend further plausibility to our results. Our findings will require replication in future studies, particularly in diet and cancer studies of prospective design with adequate assessment of meat-cooking methods. In light of our varied findings by race and smoking status, it would be useful to integrate urinary biomarkers of BaP and other PAH metabolites (Peters *et al*, 2004; Campo *et al*, 2009; Hecht *et al*, 2010) into aetiological studies of RCC.

## Figures and Tables

**Table 1:** Dietary characteristics of participants: (a) The Kidney Cancer Study (United States), 2003–2007; (b) all combined and by race

	**Cases (*N*=1192)**		**Controls (*N*=1175)**	
**Characteristic**	***N*** a	***%*** b	***N*** a	***%*** b
**(a)**				
*Age group (years)*				
20–44	143	10.6	171	10.4
45–54	279	21.8	265	21.9
55–64	360	29.5	642	29.4
65–74	288	26.7	313	27.0
75+	105	11.5	101	11.2
				
Male	697	62.0	661	61.1
				
*Study center*				
Chicago	194	16.9	188	17.0
Detroit	981	83.1	1004	83.0
				
*Education*				
Less than high school	189	16.4	145	11.3
High school graduate	405	34.5	376	31.3
Some college (1–3 years)	316	26.2	351	27.8
College graduate	265	22.8	320	29.6
				
*Smoking status*				
Never	417	35.3	458	38.4
Occasional	53	4.7	52	3.9
Regular, former	398	34.8	432	38.3
Regular, current	307	25.1	250	19.4
				
*Body mass index (kg* *m*^*−2*^*)*				
<25	234	19.4	352	28.8
25–<30	420	36.9	481	42.0
30–<35	298	25.2	210	18.0
35+	216	17.6	142	10.8
				
History of hypertension	666	57.5	486	40.3
				
*Family history of cancer* c				
None	499	42.5	545	45.7
Cancer of the kidney	50	4.3	23	1.6
Other cancer	616	52.4	614	55.5
				

	**All participants**				**Caucasians**				**African Americans**			
	**Cases (*N*=1192)**		**Controls (*N*=1175)**		**Cases (*N*=840)**		**Controls (*N*=703)**		**Cases (*N*=335)**		**Controls (*N*=489)**	
**Dietary intake, daily** d	**Mean** b	**s.e.** b	**Mean** b	**s.e.** b	**Mean** b	**s.e.** b	**Mean** b	**s.e.** b	**Mean** b	**s.e.** b	**Mean** b	**s.e.** b
**(b)**												
Total energy intake (kcal)	2375	25.5	2330	26.0	2288	25.8	2261	27.1	2634	57.9	2538	68.7
Total meat	72.6	0.8	72.1	0.8	71.0	0.9	71.7	0.9	77.1	1.5	75.1	1.3
												
*White meat*	30.1	0.6	31.2	0.6	28.0	0.6	29.3	0.7	36.2	1.3	37.0	1.0
Poultry	19.5	0.5	20.2	0.5	17.9	0.5	18.9	0.6	24.4	1.4	24.1	0.8
Fish	10.6	0.3	11.0	0.3	10.2	0.3	10.4	0.3	11.8	0.4	12.9	0.5
												
*Red meat*	42.5	0.6	40.8	0.6	43.0	0.7	41.8	0.7	40.9	0.9	38.1	1.0
Not processed	28.5	0.5	27.4	0.5	29.8	0.5	28.7	0.6	24.9	0.7	23.6	0.7
Processed	13.9	0.2	13.4	0.3	13.3	0.3	13.1	0.4	15.9	0.5	14.5	0.6
												
Barbecued meat	5.2	0.3	4.7	0.2	6.3	0.4	5.5	0.2	2.1	0.3	2.0	0.3
Pan-fried meat	6.5	0.2	6.2	0.3	5.2	0.2	4.9	0.3	10.5	0.5	10.3	0.6
Broiled meat	3.0	0.2	3.3	0.2	3.2	0.2	3.3	0.3	2.5	0.3	3.2	0.3
BaP (ng)	31.1	1.8	25.9	1.4	34.3	2.4	29.0	1.8	21.2	2.0	16.4	1.3
DiIMeIQx (ng)	2.1	0.1	1.9	0.1	1.8	0.1	1.6	0.1	3.0	0.3	2.6	0.2
MeIQx (ng)	39.3	1.4	34.5	1.1	34.1	1.5	29.4	1.3	54.9	3.1	49.8	2.8
PhIP (ng)	123.2	6.0	104.4	4.8	105.5	6.6	89.5	4.4	176.4	15.1	149.4	12.7
Alcohol (g)	234.7	14.4	195.6	13.3	217.6	16.2	190.3	15.1	286.2	38.3	212.9	28.5
Fruit and vegetables, MPED^e^	1.4	0.01	1.5	0.01	1.4	0.02	1.5	0.02	1.5	0.03	1.5	0.03

Abbreviations: BaP=benzo(*a*)pyrene; DiMeIQx=2-amino-3,4,8-trimethylimidazo[4,5-*f*]quinoxaline; MPED=MyPyramid Equivalent Database value; PhIP=2-amino-1-methyl-6-phenyl-imidazo[4,5-*b*]pyridine.

a Unweighted frequency, excludes missing/unknown/don’t know/other.

b Incorporates post-stratified weights.

c First-degree relative ever diagnosed.

d Standardised for total non-alcohol energy intake; presented as g per 1000 kcal, unless otherwise stated.

e MyPyramid food group, ounce equivalents per day, per 1000 kcal.

**Table 2:** Correlations^a^ between meat-related exposures in controls, The Kidney Cancer Study (United States), 2003–2007

	**Heterocyclic amines**			**Cooking method**				**Meat type**		
	**DiMeIQx**	**MeIQx**	**PhIP**	**Barbecue**	**Pan-fry**	**Broil**	**Other**	**Red, np** b	**White**	**Process**
BaP	NS	0.21	0.38	0.73	0.08	−0.11	0.08	0.47	0.22	0.18
DiMeIQx		0.80	0.45	0.11	0.55	NS	NS	0.38	0.11	0.17
MeIQx			0.57	0.13	0.77	NS	NS	0.54	0.22	0.36
PhIP				0.25	0.55	0.09	NS	0.37	0.29	0.23
Barbecue					−0.06	−0.14	NS	0.38	0.13	0.08
Pan-fry						NS	NS	0.45	0.28	0.41
Broil							NS	0.21	0.13	NS
Other								0.27	0.31	0.14
Red, np^b^									0.33	0.44
White										0.28

Abbreviations: BaP=benzo(*a*)pyrene; DiMeIQx=2-amino-3,4,8-trimethylimidazo[4,5-*f*]quinoxaline; MeIQx=2-amino-3,8-dimethylimidazo[4,5-*f*]quinoxaline; PhIP=2-amino-1-methyl-6-phenyl-imidazo[4,5-*b*]pyridine.

a Only statistically significant Pearson's correlation is shown (*P*<0.05); otherwise, indicated as not significant (NS).

b Red meat, not including processed; all processed meat assumed to be red; thus, white meat does not include processed.

**Table 3:** ORs and 95% CIs for renal cell carcinoma and meat-related exposures, The Kidney Cancer Study (United States), 2003–2007

**Dietary intake** a	**Cases**	**Controls**	**Median** b	**OR** c	**95% CI** c	***P*_trend_**
*Total meat* a						
Q1	306	298	45.7	1.00		
Q2	278	298	62.8	0.89	0.69, 1.16	
Q3	297	298	78.9	0.97	0.75, 1.25	
Q4	300	298	100.9	0.96	0.73, 1.26	0.91
						
*Red meat* a						
Q1	230	298	11.7	1.00		
Q2	281	298	20.4	1.03	0.78, 1.36	
Q3	300	298	28.8	0.99	0.76, 1.30	
Q4	364	298	42.0	1.11	0.83, 1.48	0.51
						
*White meat* a						
Q1	310	298	13.5	1.00		
Q2	349	298	23.4	1.31	1.02, 1.67	
Q3	269	298	34.8	1.16	0.91, 1.49	
Q4	247	298	54.8	1.11	0.87, 1.42	0.77
						
*Intake of meat cooked by different methods*						
Barbecued meat^a^						
Q1	591	682	0	1.00		
Q2	164	168	2.7	1.09	0.81, 1.48	
Q3	206	169	6.4	1.31	1.00, 1.72	
Q4	214	173	16.7	1.35	1.01, 1.79	0.03
Pan-fried meat^a^						
Q1	258	298	0.3	1.00		
Q2	313	298	2.1	1.12	0.88, 1.43	
Q3	333	298	6.0	1.32	1.03, 1.68	
Q4	271	298	15.6	1.05	0.80, 1.38	0.96
Broiled meat^a^						
Q1	520	472	0	1.00		
Q2	124	124	0.7	1.01	0.73, 1.41	
Q3	291	298	2.2	0.85	0.67, 1.07	
Q4	240	298	7.6	0.75	0.59, 0.96	0.03
						
*PAH intake from meat*						
BaP (ng per day)						
Q1	246	298	1.5	1.00		
Q2	251	298	4.3	1.06	0.80, 1.42	
Q3	291	298	11.3	1.13	0.86, 1.49	
Q4	387	298	58.3	1.50	1.14, 1.95	0.001
						
*HCA intake from meat*						
DiMeIQx (ng per day)						
Q1	286	298	0	1.00		
Q2	296	298	0.5	0.87	0.67, 1.13	
Q3	277	298	1.3	0.82	0.56, 1.12	
Q4	316	298	3.9	0.94	0.69, 1.27	0.81
MeIQx (ng per day)						
Q1	281	298	6.2	1.00		
Q2	291	298	16.7	1.03	0.80, 1.32	
Q3	281	298	32.7	1.04	0.79, 1.37	
Q4	322	298	73.3	1.11	0.80, 1.55	0.39
PhIP (ng per day)						
Q1	270	298	3.7	1.00		
Q2	288	298	23.0	0.95	0.70, 1.27	
Q3	309	298	73.2	0.91	0.67, 1.25	
Q4	308	298	249.9	0.92	0.68, 1.26	0.88

Abbreviations: BaP=benzo(*a*)pyrene; BMI, body mass index; CI=confidence interval; DiMeIQx=2-amino-3,4,8-trimethylimidazo[4,5-*f*]quinoxaline; HCA=heterocyclic amine; OR=odds ratio; PAH=polycyclic aromatic hydrocarbon; PhIP=2-amino-1-methyl-6-phenyl-imidazo[4,5-*b*]pyridine; Q=quartile.

a Daily dietary intake adjusted for total non-alcohol energy intake; presented as g per 1000 kcal, unless otherwise specified; red and white meat do not include processed.

b Median intake value for each Q based on distribution among all study controls.

c Models adjusted for age, race, sex, education, smoking status, BMI, history of hypertension, family history of cancer, intake of alcohol, intake of fruit and vegetables, total non-alcohol energy intake, and other meat intake and/or cooking method offsets.

**Table 4:** ORs and 95% CIs for renal cell carcinoma and meat-cooking methods, stratified by race, The Kidney Cancer Study (United States), 2003–2007

	**Caucasians**						**African Americans**					
**Dietary intake** a	**Cases**	**Controls**	**Median** b	**OR** c	**95% CI** c	***P*_trend_**	**Cases**	**Controls**	**Median** b	**OR** c	**95% CI** c	***P*_trend_**
*Intake of meat cooked by different methods*												
Barbecued meat^a^												
Q1	355	317	0	1.00			236	365	0	1.00		
Q2	122	114	2.8	1.05	0.74, 1.49		42	54	2.5	1.34	0.78, 2.33	
Q3	167	130	6.4	1.29	0.95, 1.74		39	39	5.9	1.62	0.94, 2.78	
Q4	196	142	16.8	1.44	1.05, 1.97	0.02	18	31	16.3	0.92	0.48, 1.78	0.52
Pan-fried meat^a^												
Q1	210	217	0.4	1.00			48	81	0.1	1.00		
Q2	266	213	2.1	1.21	0.92, 1.58		47	85	2.2	0.89	0.47, 1.69	
Q3	231	166	5.6	1.40	1.05, 1.88		102	132	6.7	1.24	0.77, 2.00	
Q4	133	107	14.7	1.10	0.77, 1.58	0.78	138	191	16.7	1.05	0.65, 1.72	0.87
Broiled meat^a^^,^^d^												
Q1	368	282	0	1.00			152	190	0	1.00		
Q2	81	66	0.7	0.97	0.64, 1.46		43	58	0.6	1.08	0.63, 1.87	
Q3	210	180	2.3	0.85	0.64, 1.13		81	118	2.2	0.89	0.58, 1.37	
Q4	181	175	8.0	0.84	0.62, 1.15	0.30	59	123	7.3	0.55	0.35, 0.85	0.004
												
*PAH intake from meat*												
BaP (ng per day)^f^												
Q1	188	181	1.4	1.00			58	117	1.6	1.00		
Q2	169	146	4.1	1.06	0.75, 1.49		82	152	4.4	1.32	0.81, 2.16	
Q3	170	157	11.1	0.97	0.68, 1.38		121	141	12.1	1.77	1.10, 2.83	
Q4	313	219	60.8	1.42	1.04, 1.93	0.003	74	79	46.6	2.00	1.15, 3.47	0.08
												
*HCA intake from meat*												
DiMeIQx (ng per day)												
Q1	214	184	0.03	1.00			72	114	0.03	1.00		
Q2	222	488	0.5	0.82	0.61, 1.11		74	110	0.5	0.96	0.60, 1.56	
Q3	194	179	1.2	0.76	0.54, 1.09		83	119	1.3	0.86	0.54, 1.36	
Q4	210	152	3.7	0.99	0.72, 1.36	0.58	106	146	4.6	0.81	0.53, 1.25	0.37
MeIQx (ng per day)												
Q1	220	202	6.6	1.00			61	96	5.4	1.00		
Q2	237	212	16.5	0.94	0.72, 1.24		54	86	17.3	1.13	0.68, 1.89	
Q3	203	170	32.7	0.97	0.73, 1.29		78	128	32.6	0.89	0.55, 1.44	
Q4	180	119	68.4	1.15	0.77, 1.72	0.38	142	179	78.7	1.04	0.67, 1.61	0.92
PhIP (ng per day)												
Q1	171	154	4.2	1.00			99	144	3.4	1.00		
Q2	227	193	22.6	1.00	0.73, 1.37		61	105	23.6	0.80	0.49, 1.32	
Q3	243	203	73.6	1.01	0.73, 1.40		66	95	72.8	0.72	0.46, 1.12	
Q4	199	153	221.6	1.05	0.78, 1.42	0.70	109	145	281.2	0.86	0.51, 1.42	0.83

Abbreviations: BaP=benzo(*a*)pyrene; BMI=body mass index; CI=confidence interval; DiMeIQx=2-amino-3,4,8-trimethylimidazo[4,5-*f*]quinoxaline; HCA=heterocyclic amine; OR=odds ratio; PAH=polycyclic aromatic hydrocarbon; PhIP=2-amino-1-methyl-6-phenyl-imidazo[4,5-*b*]pyridine; Q=quartile.

a Daily dietary intake adjusted for total non-alcohol energy intake; presented as g per 1000 kcal, unless otherwise specified.

b Median intake value for each Q based on distribution among all study controls.

c Models adjusted for age, sex, education, smoking status, BMI, history of hypertension, family history of cancer, intake of alcohol, intake of fruit and vegetables, total non-alcohol energy intake, and other meat intake and/or cooking method offset.

d Significant interaction *P*_broil × race_=0.03.

e Models adjusted for age, sex, education, smoking status, BMI, history of hypertension, family history of cancer, intake of alcohol, intake of fruit and vegetables, and total non-alcohol energy intake.

f Significant interaction *P*_BaP × race_=0.05.

**Table 5:** ORs and 95% CIs for renal cell carcinoma and BaP intake, stratified by smoking status^a^, The Kidney Cancer Study (United States), 2003–2007

	**Never smokers**					**Current-regular smokers**				
	**Q1**	**Q2**	**Q3**	**Q4**	***P*_trend_**	**Q1**	**Q2**	**Q3**	**Q4**	***P*_trend_**
OR^b^	1.00	1.24	1.01	1.47	0.12	1.00	1.28	1.95	2.63	0.006
95% CI	–	(0.77, 1.98)	(0.63, 1.72)	(0.93, 2.31)		–	(0.68, 2.42)	(1.00, 3.87)	(1.36, 5.10)	
Cases/controls	93/116	95/96	102/125	127/121		46/55	62/71	85/64	114/60	
Median^c^ (ng per day)	1.5	4.2	11.1	58.4		1.3	4.1	12.2	52.8	

Abbreviations: BaP=benzo(*a*)pyrene; CI=confidence interval; OR=odds ratio; Q=quartile.

a Significant interaction *P*_BaPxsmoke_=0.02; no association in former/occasional smokers (*P*_trend_=0.10).

b Models adjusted for age, race, sex, education, BMI, history of hypertension, family history of cancer, intake of alcohol, intake of fruit and vegetables, and total non-alcohol energy intake.

c Median intake value for each Q based on distribution among all study controls.
