# Supplementary material for: Meat-cooking mutagens and risk of renal cell carcinoma
Source: Br J Cancer. 2011 Sep 6;105(7):1096–104. doi: 10.1038/bjc.2011.343 (PMC3185955; doi:10.1038/bjc.2011.343)
Supplement: Supplementary Table S1 [file bjc2011343x1.doc]

Supplement: Intake of meat cooked by different methods from red and white meat sources

|  | **Red meat sources** | | | | | **White meat sources** | | | | |
| --- | --- | --- | --- | --- | --- | --- | --- | --- | --- | --- |
|  | median (controls) | ca/co | OR | 95% CI | P_trend_ | median (controls) | ca/co | OR | 95% CI | P_trend_ |
| ***Cooking methods^*^*** |  |  |  |  |  |  |  |  |  |  |
| **Barbecued, g** |  |  |  |  |  |  |  |  |  |  |
| Q1 | 0.0 | 621/704 | 1.00 |  |  | 0.0 | 1041/1081 | 1.00 |  |  |
| Q2 | 5.7 | 174/169 | 1.13 | 0.83-1.53 |  | 9.2 | 53/37 | 1.42 | 0.87-2.32 |  |
| Q3 | 13.1 | 179/157 | 1.28 | 0.98-1.65 |  | 20.0 | 42/38 | 0.80 | 0.46-1.28 |  |
| Q4 | 28.5 | 201/162 | 1.31 | 1.00-1.73 | 0.13 | 30.4 | 39/36 | 1.19 | 0.69-2.08 | 0.38 |
| **Pan-fried, g** |  |  |  |  |  |  |  |  |  |  |
| Q1 | 0.0 | 66/85 | 1.00 |  |  | 0.0 | 1031/1032 | 1.00 |  |  |
| Q2 | 1.7 | 332/366 | 0.98 | 0.66-1.47 |  | 13.3 | 56/62 | 0.97 | 0.61-1.55 |  |
| Q3 | 7.9 | 395/364 | 1.22 | 0.83-1.80 |  | 27.5 | 36/44 | 0.90 | 0.54-1.50 |  |
| Q4 | 25.3 | 382/377 | 1.17 | 0.78-1.77 | 0.19 | 42.9 | 52/54 | 1.09 | 0.68-1.77 | 0.94 |
| **Broiled, g** |  |  |  |  |  |  |  |  |  |  |
| Q1 | 0.0 | 542/499 | 1.00 |  |  | 0.0 | 1098/1099 | 1.00 |  |  |
| Q2 | 2.6 | 255/278 | 0.93 | 0.72-1.19 |  | 7.7 | 26/31 | 0.78 | 0.43-1.39 |  |
| Q3 | 4.5 | 172/186 | 0.86 | 0.64-1.15 |  | 13.3 | 23/31 | 0.81 | 0.44-1.48 |  |
| Q4 | 18.4 | 206/229 | 0.91 | 0.71-1.17 | 0.66 | 30.1 | 28/31 | 1.13 | 0.63-2.01 | 0.6 |
| **Other cook^**^, g** |  |  |  |  |  |  |  |  |  |  |
| Q1 | 0.0 | 667/675 | 1.00 |  |  | 0.0 | 154/188 | 1.00 |  |  |
| Q2 | 2.8 | 177/171 | 1.15 | 0.83-1.59 |  | 7.8 | 305/332 | 1.11 | 0.81-1.50 |  |
| Q3 | 6.1 | 204/186 | 1.13 | 0.84-1.54 |  | 18.3 | 331/334 | 1.10 | 0.85-1.43 |  |
| Q4 | 21.2 | 127/160 | 1.25 | 0.93-1.68 | 0.54 | 30.6 | 385/338 | 0.98 | 0.72-1.35 | 0.86 |
| ^*^ Set reference to zero (remainder categorized into tertiles) for cooking methods by red and white meat source for the sake of comparison | | | | | | | | | | |
| ^**^ Baked, stewed, or microwaved | |  |  | | | | | | | |

Supplement continued: Intake of meat-cooking mutagens from red and white meat sources

|  | **Red meat sources** | | | | | **White meat sources** | | | | |
| --- | --- | --- | --- | --- | --- | --- | --- | --- | --- | --- |
|  | median (controls) | ca/co | OR | 95% CI | P_trend_ | median (controls) | ca/co | OR | 95% CI | P_trend_ |
| ***Meat-cooking mutagens*** | |  |  |  |  |  |  |  |  |  |
| **B[*a*]P, ng** |  |  |  |  |  |  |  |  |  |  |
| Q1 | 0.6 | 225/298 | 1.00 |  |  | 0.1 | 327/311 | 1.00 |  |  |
| Q2 | 2.5 | 271/298 | 1.14 | 0.86-1.52 |  | 0.5 | 279/285 | 1.00 | 0.79-1.27 |  |
| Q3 | 7.4 | 309/298 | 1.25 | 0.96-1.64 |  | 1.5 | 300/291 | 1.03 | 0.80-1.33 |  |
| Q4 | 50.1 | 370/298 | 1.50 | 1.17-1.92 | 0.001 | 4.9 | 278/296 | 1.02 | 0.80-1.30 | 0.99 |
| *continuous, per 5 ng* |  |  | *1.01* | *1.00-1.02* |  |  |  | *1.02* | *0.99-1.05* |  |
| **PhIP, ng** |  |  |  |  |  |  |  |  |  |  |
| Q1 | 2.1 | 259/298 | 1.00 |  |  | 0.0 | 820/828 | 1.00 |  |  |
| Q2 | 20.3 | 241/298 | 0.86 | 0.67-1.11 |  | 30.2 | 115/122 | 0.95 | 0.70-1.28 |  |
| Q3 | 41.4 | 321/298 | 1.07 | 0.83-1.39 |  | 104.8 | 123/119 | 0.96 | 0.70-1.31 |  |
| Q4 | 116.6 | 354/298 | 1.17 | 0.91-1.51 | 0.16 | 284.4 | 117/123 | 1.02 | 0.76-1.36 | 0.98 |
| *continuous, per 20 ng* |  |  | *1.02* | *1.00-1.04* |  |  |  | *1.00* | *0.99-1.02* |  |
| **DiMeIQx^***^, ng** |  |  |  |  |  |  |  |  |  |  |
| Q1 | 0.0 | 264/302 | 1.00 |  |  |  |  |  |  |  |
| Q2 | 0.3 | 309/297 | 0.96 | 0.72-1.27 |  |  |  |  |  |  |
| Q3 | 1.0 | 294/295 | 0.93 | 0.71-1.21 |  |  |  |  |  |  |
| Q4 | 3.2 | 308/298 | 0.95 | 0.72-1.25 | 0.96 |  |  |  |  |  |
| *continuous, per 1 ng* |  |  | *1.00* | *0.98-1.02* |  |  |  | *1.05* | *0.98-1.12* |  |
| **MeIQx, ng** |  |  |  |  |  |  |  |  |  |  |
| Q1 | 4.5 | 269/298 | 1.00 |  |  | 0.0 | 350/369 | 1.00 |  |  |
| Q2 | 14.9 | 284/298 | 0.97 | 0.75-1.26 |  | 0.5 | 228/227 | 1.07 | 0.84-1.37 |  |
| Q3 | 30.3 | 302/298 | 1.08 | 0.84-1.38 |  | 1.6 | 314/298 | 1.11 | 0.89-1.38 |  |
| Q4 | 66.1 | 320/298 | 1.13 | 0.85-1.52 | 0.66 | 7.1 | 283/298 | 1.12 | 0.89-1.42 | 0.74 |
| *continuous, per 5 ng* |  |  | *1.01* | *0.99-1.02* |  |  |  | *1.01* | *0.95-1.07* |  |
| ^***^ Primarly zero values from white meat sources; OR's for zero intake vs. any intake were null. | | | | | | |  |  |  |  |
